# Estudio de la cristaluria: efectividad de la incorporación de medidas higiénico-dietéticas en los informes de laboratorio

**DOI:** 10.1515/almed-2020-0089

**Published:** 2021-01-11

**Authors:** Paula Sienes Bailo, María Santamaría González, Silvia Izquierdo Álvarez, Raquel Lahoz Alonso, Patricia Serrano Frago, José Luis Bancalero Flores

**Affiliations:** Servicio de Bioquímica Clínica, Hospital Universitario Miguel Servet, Zaragoza, España; Servicio de Urología, Hospital Universitario Miguel Servet, Zaragoza, España

**Keywords:** cólico renal, nefrolitiasis, cálculos, recomendaciones higiénico-dietéticas

## Abstract

**Objetivos:**

El presente estudio evalúa la efectividad de incorporar medidas higiénico-dietéticas en los informes de laboratorio comparando la incidencia de cólicos nefríticos (CN) en dos grupos de pacientes que presentaron, al menos, un evento de cristaluria asociada con riesgo litogénico, indicando solo a uno de ellos la introducción de estas recomendaciones.

**Métodos:**

Estudio observacional retrospectivo. Grupo A: 1.115 pacientes que en el año 2017 presentaron al menos un episodio de cristaluria asociada con riesgo litogénico y no recibieron recomendaciones higiénico-dietéticas con el informe de laboratorio. Grupo B: 1.692 pacientes que en el año 2018 presentaron al menos un episodio de cristaluria asociada con riesgo litogénico y recibieron recomendaciones higiénico-dietéticas con el informe de laboratorio. Las diferencias entre grupos según sexo, edad y tipo de cristales urinarios fueron analizadas mediante pruebas χ^2^ y U de Mann–Whitney.

**Resultados:**

La incidencia de CN fue de 2,24% en el grupo A *v* 1,12% en el grupo B. Los pacientes del grupo A presentaron 2,02 veces más CN que los del grupo B. No se encontraron diferencias significativas en la incidencia de CN según el tipo de cristal urinario. La incidencia de CN en pacientes que presentaron al menos un evento de cristaluria asociada con riesgo litogénico durante el año de estudio fue mucho mayor que la de la población general en el mismo período (0,46%, similar a las publicadas previamente).

**Conclusiones:**

La introducción de mensajes que alertan sobre el riesgo litogénico junto con la inclusión de medidas higiénico-dietéticas en los informes de laboratorio son herramientas para tratar de reducir la incidencia de CN.

## Introducción

El cólico nefrítico (CN) es la presentación clínica más frecuente de la litiasis renal. En España, se estima que el 10–20% de los varones y el 3–5% de las mujeres sufrirán, al menos, un episodio de CN a lo largo de su vida y el 30–40% de los mismos podrán sufrir una recidiva en los siguientes 5 años. Los hombres con edades comprendidas entre 30 y 60 años son, en general, el grupo más afectado [1]. La frecuencia de CN aumenta durante los meses cálidos y en las primeras horas de la mañana, hallándose ligada a variaciones geográficas en función de la dieta y la ingesta de agua [2]. En cuanto a la composición, la litiasis oxalocálcica es la más frecuente, seguida de la úrica, mixta (oxalato y fosfato de calcio), estruvita y cistina [3].

La cristaluria es más frecuente en pacientes litiásicos que en sujetos sanos, aunque ocasionalmente pueden encontrarse cristales en la orina de individuos sin esta patología. El análisis del sedimento urinario es una herramienta valiosa para la detección y monitorización de enfermedades hereditarias y adquiridas asociadas a la formación de cálculos urinarios, ya que la cristaluria aumenta sustancialmente la probabilidad de formación de los mismos [4], [5], [6]. En esta línea, la guía de la *American Urological Association* (AUA) para el manejo de los cálculos renales recomienda que el análisis de orina incluya tanto la evaluación microscópica del sedimento urinario como el estudio del análisis cualitativo para evaluar el pH urinario, los indicadores de infección e identificar cristales patognomónicos de cada tipo de cálculo [7].

Determinados cristales urinarios se asocian a un mayor riesgo litogénico en base a su naturaleza química y cristalina, facies cristalina, abundancia de la cristaluria, tamaño de los cristales, tasa de agregación y maclación y frecuencia de la cristaluria. Los cristales de oxalato cálcico dihidratado (OCD, weddellita) que generalmente presentan forma octaédrica o bipiramidal, adoptan una conformación dodecaédrica [8] cuando aumentan los niveles de calcio en la orina, lo que también aumenta el riesgo litogénico por ser el calcio un promotor de la cristalización. Los cristales de OCD con un tamaño entre 7 y 8 μm pueden encontrarse en pacientes sanos, mientras que la presencia de cristales de OCD mayores de 35 μm indica litogénesis activa y mayor riesgo de recidiva. Del mismo modo, la litiasis úrica se asocia con la presencia de cristales de ácido úrico (AU) mayores de 100 μm. Finalmente, la presencia en el mismo sedimento urinario de cristales de oxalato cálcico monohidratado (OCM, wewellita) y dihidratado, relacionada con hiperoxaluria e hipercalciuria simultáneas, aumenta el riesgo de litiasis [9].

En los últimos años, algunos investigadores han querido incidir en la relación que se establece entre la urolitiasis y la alimentación, en particular con la baja ingesta de agua y con el consumo de dietas ricas en proteínas animales, azúcares, oxalato y sodio [10], [11]. Por ello, la idea de dirigir recomendaciones higiénico-dietéticas a pacientes con riesgo litogénico se propone como una medida adecuada para reducir la incidencia de esta patología.

El presente estudio evalúa la efectividad de incorporar medidas higiénico-dietéticas en los informes de laboratorio comparando la incidencia de CN en dos grupos de pacientes atendidos en el Hospital Universitario Miguel Sevet (HUMS) durante los años 2017 y 2018 y que presentaron al menos un evento de cristaluria asociada con riesgo litogénico (AU >100 μm, OCD >35 μm, OCD dodecaédrico y presencia simultánea de OCD y OCM), indicando solo en uno de ellos la introducción de estas recomendaciones.

## Materiales y métodos

### Sujetos y materiales

Estudio observacional retrospectivo. Los datos se obtuvieron de los registros del Sistema Informático de laboratorio (Modulab, Werfen, España) y de la historia clínica electrónica de los pacientes, revisada por los facultativos de la Sección de Función Renal del HUMS, Zaragoza, España en 2019.

**Figura 1:**
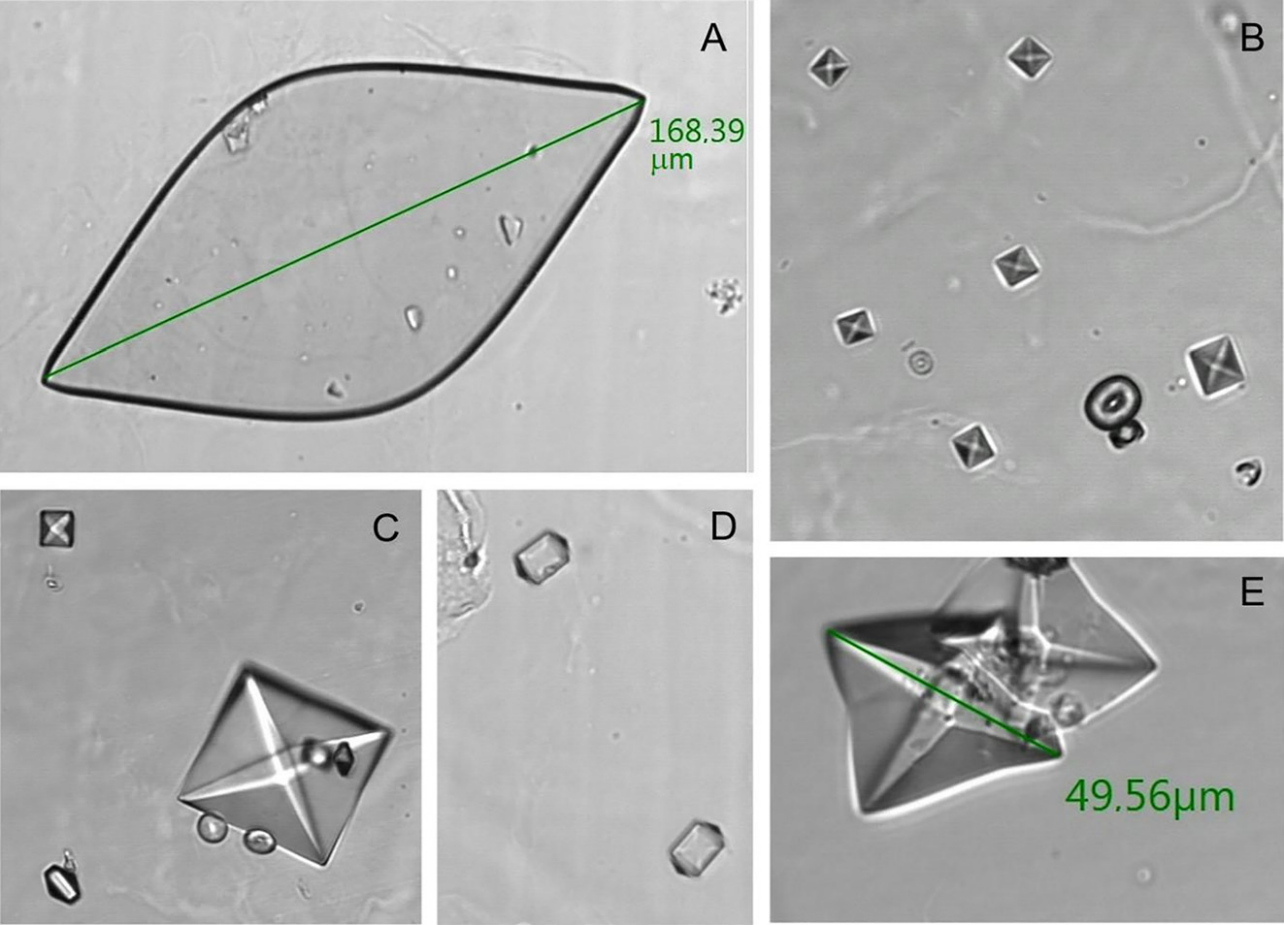
(A–E) Imágenes SEDIMAX (A. Menarini Diagnostics, Barcelona, España). (A) Cristales de ácido úrico (AU) >100 μm. (B) Cristales de oxalato cálcico monohidratado (OCM) y dihidratado (OCD) en el mismo sedimento urinario. (C) Cristales de OCD >35 μm y dodecaédricos en el mismo sedimento urinario. (D) Cristales de OCD dodecaédricos. (E) Cristales de OCD >35 μm.

Criterios de inclusión: pacientes estudiados en el HUMS durante 2017 (grupo A) y 2018 (grupo B) definidos como población con riesgo litogénico. La identificación de los sujetos del estudio como una población con riesgo litogénico requiere que los pacientes se hayan realizado, al menos, un análisis del sedimento urinario en 2017 o 2018 y que cumplan una (Figura 1A, B, D, E) o más (Figura 1C) de las siguientes condiciones [9]:

- Presencia de cristales de AU mayores de 100 μm (Figura 1A)
- Presencia de cristales de OCD mayores de 35 μm (Figura 1C, E)
- Presencia de cristales de OCD dodecaédricos (Figura 1C, D)
- Presencia en el mismo sedimento urinario de cristales de OCD y OCM (Figura 1B)

Los sujetos fueron divididos en dos grupos según sus informes de laboratorio incluyeran o no un comentario que alertara de la presencia de riesgo litogénico y una serie de recomendaciones higiénico-dietéticas que se proponían al médico peticionario para la prevención de los CN:

– Grupo A. Pacientes con riesgo litogénico a los que no se adjuntó un comentario de alerta sobre la detección del riesgo litogénico ni la recomendación de implementar ciertas medidas higiénico-dietéticas en su informe de laboratorio. Este grupo estaba compuesto por pacientes del Sector Zaragoza II que en 2017 presentaron al menos un episodio de cristaluria asociado con riesgo litogénico.
– Grupo B. Pacientes con riesgo litogénico que recibieron un comentario de alerta sobre la detección del riesgo litogénico y la recomendación de implementar las medidas higiénico-dietéticas incluidas en su informe de laboratorio (Tabla 1). Este grupo estaba formado por pacientes del Sector Zaragoza II que en 2018 presentaron al menos un episodio de cristaluria asociado con riesgo litogénico.

**Tabla 1:** Comentarios de alarma: riesgo litogénico (A) y recomendaciones higiénico-dietéticas incluidas (B) en los informes de laboratorio.

(A)	**Comentarios de alarma: riesgo litogénico**
1.	Riesgo litogénico. Presencia de algunos cristales de ácido úrico de tamaño >100 µm. Valorar litiasis y/o alteración metabólica. Valorar implementar medidas higiénico-dietéticas.
2.	Riesgo litogénico. Presencia de algunos cristales de oxalato cálcico de tamaño >35 µm. Litogénesis activa. Valorar implementar medidas higiénico-dietéticas.
3.	Riesgo litogénico. Presencia de cristales de oxalato cálcico dodecaédricos. Valorar implementar medidas higiénico-dietéticas.
4.	Riesgo litogénico. Presencia de cristales de oxalato cálcico monohidrato y dihidrato. Valorar implementar medidas higiénico-dietéticas.
**(B)**	**Recomendaciones higiénico-dietéticas**
–	Aumentar la ingesta de líquidos a 2–3 L agua/día.
–	Aumentar el consumo de alimentos ricos en fitato y citrato.
–	Mantener el consumo de alimentos ricos en calcio.
–	Disminuir el consumo de alimentos ricos en oxalato, sal, proteína animal y refrescos.
–	Evitar suplementos de calcio y vitaminas C y D.
–	Evitar el sedentarismo.
(Consulte con su médico antes de dejar de tomar cualquier medicación prescrita).	

Criterios de exclusión. Pacientes pertenecientes a otro Sector Sanitario, pacientes que no se realizaron un análisis del sedimento urinario en 2017 o 2018 y aquellos que no cumplieran al menos una de las condiciones antes mencionadas asociadas con un mayor riesgo litogénico.

### Métodos y técnicas

Muestras de la primera orina matinal recogidas por el paciente en tubos VACUETTE^®^ de 10 mL (Greiner bio-one, Madrid, España) y remitidas al HUMS. Allí, los facultativos de la Sección de Función Renal realizaron el análisis del sedimento urinario, usando en tándem los analizadores AutionMax AX-4030 y SediMax (A. Menarini Diagnostics, Barcelona, España). El primero de ellos realiza automáticamente un primer cribado con tiras reactivas de orina. Si alguna magnitud aparece alterada en estas, SediMax realiza el sedimento urinario aspirando 2 mL de muestra y transfiriendo 0,2 mL a una cubeta donde se centrifuga a 2.000 rpm durante 10 minutos. Después, un microscopio de campo brillante toma quince fotografías a 400 aumentos en diferentes campos. Finalmente, las imágenes son revisadas por los facultativos de la Sección de Función Renal, quienes identifican, miden y cuantifican los diferentes elementos de la orina.

Los pacientes que cumplieron cualquiera de los cuatro criterios asociados con un mayor riesgo litogénico se incluyeron en el grupo A o en el B según el año de estudio. Los pacientes estudiados en 2017 fueron incluidos en el grupo A, no recibieron ningún comentario adjunto a su informe de laboratorio y fueron seguidos durante el año 2018 para anotar cuántos sufrieron un evento de CN. Por otro lado, los estudiados en 2018 formaron el grupo B, recibieron junto con el informe de laboratorio un comentario de alerta dirigido al médico peticionario sobre la presencia de riesgo litogénico y una serie de medidas higiénico-dietéticas recomendadas para el paciente (Tabla 1) y fueron seguidos durante el año 2019 para anotar el número de CN que se produjeron. De esta forma, el tiempo de seguimiento de ambos grupos fue el mismo e igual a un año completo.

La incidencia de CN en la población general se calculó a partir del número total de CN atendidos en el Servicio de Urgencias del HUMS en 2017 y 2018 y la cifra de población asignada al Sector Sanitario Zaragoza II en esos años, proporcionada por el Departamento de Gestión de Sistemas de Información del HUMS. Por otro lado, se calculó la incidencia de CN en el grupo A y B atendiendo al número de CN sufridos por los pacientes de cada grupo y tratados por este mismo Servicio.

Este estudio cumple con todas las regulaciones nacionales, políticas institucionales y principios éticos de la Declaración de Helsinki, y fue aprobado por el Comité de Ética en Investigación de la Comunidad Autónoma de Aragón (CEICA).

### Análisis estadísticos

Se calculó la distribución de frecuencias por categorías para cada variable cualitativa. La edad, como única variable cuantitativa fue explorada con la prueba de Shapiro-Wilk (bondad de ajuste a una distribución normal) y se calcularon indicadores de tendencia central (mediana) y dispersión (rango intercuartílico, RIC). Las diferencias entre los grupos según sexo, edad, incidencia de CN y tipo de cristales urinarios fueron analizadas mediante pruebas de contraste de hipótesis con comparación de proporciones (χ^2^) y medianas (U de Mann-Whitney). Los datos se analizaron utilizando el software estadístico Jamovi 1.1.9.0 y la significación estadística se determinó en p<0,05 (dos colas).

El análisis estadístico se completó con la construcción de un modelo de regresión, tomando como variable dependiente la presencia o ausencia de CN y como independientes aquellas que presentaron diferencias estadísticamente significativas en las pruebas de contraste de hipótesis.

## Resultados

De 192.024 urianálisis, se realizaron 84.342 sedimentos urinarios en 2017 y 1.115 pacientes fueron incluidos en el grupo A por cumplir, al menos, uno de los cuatro criterios asociados con un mayor riesgo litogénico. Con los mismos criterios de inclusión y exclusión, el grupo B se construyó con 1.692 pacientes analizados en 2018, de un total de 196.195 urianálisis y 89.208 sedimentos urinarios realizados ese año. En el grupo A, 25/1.115 (2,24%) pacientes sufrieron un episodio de CN ese año, como sucedió en 19/1.692 (1,12%) pacientes del grupo B. Los datos demográficos y los tipos de cristales urinarios de los pacientes se recogen en la Tabla 2.

**Tabla 2:** Datos demográficos y tipos de cristales urinarios de los pacientes estudiados.

	Grupo A 1.115	Grupo B 1.692	p-Valor
Sexo			0,311
Hombres	427 (38,3)	616 (36,4)	
Mujeres	688 (61,7)	1076 (63,6)	
Edad de aparición, años	50,0 ± 25,0	51,0 ± 27,0	0,967
Franja etaria, años			0,467
<13	15 (1,4)	29 (1,7)	
14–29	103 (9,2)	190 (11,2)	
30–45	334 (30,0)	489 (28,9)	
46–64	443 (39,7)	627 (37,1)	
>65	220 (19,7)	357 (21,1)	
Cristales urinarios			0,017^a^
AU>100, μm	96 (8,6)	126 (7,5)	
OCD>35, μm	103 (9,3)	129 (7,6)	
OCD dodecaédricos	472 (42,3)	664 (39,2)	
OCD + OCM	444 (39,8)	773 (45,7)	

Grupo A: pacientes sin comentarios de alarma ni recomendaciones higiénico-dietéticas adjuntas al informe de laboratorio. Grupo B: pacientes con comentarios de alarma y recomendaciones higiénico-dietéticas adjuntas al informe de laboratorio. La edad se expresa como mediana ± RIC y el sexo, rango de edad y tipos de cristales urinarios se expresan como N (%), en términos de frecuencias absolutas y relativas. Los p-valores obtenidos en las pruebas de comparación de medianas y proporciones se recogen en la última columna, indicando con (^a^) aquellos considerados significativos. OCD, oxalato cálcico dihidratado; OCM, oxalato cálcico monohidratado; AU, ácido úrico.

Ambos grupos estuvieron formados mayoritariamente por mujeres (61,7% *v* 63,6%) sin existir diferencias estadísticamente significativas entre ellos (χ^2^=1,03; p=0,311). La mediana de la edad de presentación del evento de cristaluria para los pacientes en el grupo A fue 50 años (RIC 25,0) similar a 51 años (RIC 27,0) en el B (p=0,967). En ambos, las distribuciones por edad no se ajustaron a la distribución normal (p=0,003 *v* p≤0,001).

La distribución por tipo de cristal urinario asociado con un mayor riesgo litogénico encontrado en el sedimento urinario de los pacientes fue significativamente diferente en los dos grupos (χ^2^=10,2; p=0,017). En ambos casos, la presencia de cristales de AU >100 μm fue el evento menos encontrado, pero mientras que para el grupo A el hallazgo más repetido fue la presencia de OCD dodecaédrico, para el B fue la presencia simultánea de cristales de OCD y OCM.

En la Tabla 3A se presentan los datos de incidencia y edad promedio de presentación de CN en ambos grupos. Se encontraron diferencias estadísticamente significativas entre la incidencia de CN en el grupo A y B, de acuerdo a la hipótesis propuesta en este estudio (χ^2^=5,46; p=0,019).

**Tabla 3:** Incidencia y mediana de la edad de presentación de CN en los grupos A y B (A) y en la población general (B).

(A)	Grupo/año de estudio	CN	Individuos/población	Incidencia de CN (%)	Mediana de la edad de presentación en años (RIC)
	A	25	1.115	2,24	48,0 (19,0)
	B	19	1.692	1,12	51,0 (20,0)
**(B)**	2017	1.796	390.787	0,46	48,0 (22,0)
	2018	1.776	394.165	0,45	48,0 (23,0)

CN, cólico nefrítico. RIC, rango intercuartílico.

La población total promedio del Sector Zaragoza II atendida por el HUMS en ese período fue 392.476 personas-año, con una tasa de incidencia general de CN del 0,46%. Los datos de cada año se muestran en la Tabla 3B junto con el número total de CN atendidos por este hospital, la edad promedio de presentación del evento y la incidencia de CN expresada como porcentajes.

Se construyó un modelo de regresión logística binomial usando la variable resultado (presencia o ausencia de CN) como variable dependiente y la inclusión de recomendaciones higiénico-dietéticas como factor. La odds ratio obtenida fue de 2,02 (IC 95% 1,11–3,68). Los pacientes pertenecientes al grupo A presentaron 2,02 veces más eventos de CN que los pacientes del grupo B que sí recibieron estas recomendaciones, existiendo una asociación estadísticamente significativa entre el factor (implementación de medidas higiénico-dietéticas en pacientes con riesgo litogénico) y el efecto (incidencia de CN) estudiado.

## Discusión

En nuestro estudio se obtuvo una incidencia de urolitiasis expresada como CN de 0,46% casos por año en la población general del Sector Zaragoza II durante el período 2017–2018, siendo similar a las publicadas previamente en la literatura [3], [12], [13].

La incidencia de urolitiasis descrita en España en 2007 fue del 0,73%, con una prevalencia del 5,06% [3], aunque tres años después, en un estudio de prevalencia de la insuficiencia renal crónica en nuestro país se comunicó una prevalencia del 13,9% [12]. Del mismo modo, el estudio PreLiRenE informó una prevalencia del 14,6% [13], inferior al 16,3% descrito en un estudio similar conocido como PreLiRenA en el que se evaluó la prevalencia de litiasis renal en la población andaluza de 40 a 65 años [14]. En el estudio PreLiRenE, Aragón se situó en un rango de prevalencia entre 14 y 16% [13].

Por otro lado, si la incidencia de CN en la población general del Sector Zaragoza II en 2017 (0,46%) se compara con la calculada en la población con riesgo litogénico de este Sector en el mismo año (grupo A: 2,24%), se puede ver cómo este número aumenta casi 5 veces. En el año siguiente, la incidencia de CN en la población general del Sector se mantuvo constante (0,45%), mientras que la incidencia en la población con riesgo litogénico se redujo a la mitad (grupo B: 1,12%). Este hecho puede atribuirse al mayor control e implementación de medidas y estilos de vida saludables en pacientes en los que se informó el riesgo litogénico, medidas con las que presumiblemente fue posible frenar el proceso de precipitación o agregación de cristales que generalmente conduce a la formación de un cálculo renal.

Similar a nuestro estudio, para demostrar la relación existente entre la cristaluria y la formación de cálculos urinarios, algunos investigadores concluyeron que la cristaluria es un paso inicial importante y necesario en la formación de cálculos renales y también un indicador de esta actividad, lo que hace que una proporción importante de los pacientes que presentan cristaluria resulten ser finalmente formadores de cálculos [15]. Otros autores, en concordancia con estos hallazgos, proponen una probabilidad aumentada de formación de cálculos cuando están presentes uno o más factores de riesgo ya que conducen a la sobresaturación de la orina, formación de cristales y su posterior agregación dando lugar a los cálculos responsables de la clínica [4]. Los individuos con litiasis recurrentes presentan cristales más grandes y agregados que los pacientes no litiásicos y se ha propuesto que la cristaluria identificada en muestras de orina matinales pronostica el riesgo de formación recurrente de cálculos [16].

La relación entre la litiasis renal y la presencia de ciertas enfermedades, factores ambientales, sociodemográficos y estilos de vida ha sido ya investigada, especialmente comportamientos sedentarios, dieta inadecuada, sobrepeso, estrés y factores como el clima o el consumo de agua se han relacionado con esta patología [11], [12], [13], [17], [18]. Se debe aconsejar a los pacientes litiásicos el aumento de la ingesta de líquidos para reducir la sobresaturación de diferentes compuestos en la orina y prevenir la formación de cálculos [10], junto con la incorporación de recomendaciones dietéticas típicas, como reducir la ingesta de proteínas y el consumo de alimentos ricos en sodio y mantener una ingesta adecuada de calcio [11], [16], [19], [20], [21], [22].

Nuestro estudio presenta algunas limitaciones como son no contemplar otros factores (clínicos o no) que puedan influir en la aparición de CN en la población estudiada. Además, el análisis del sedimento urinario solo se realizó en muestras con resultados alterados en el análisis cualitativo de orina, por lo que pacientes con cristaluria que presentaron resultados normales en este primer método de detección pudieron haberse perdido. Por otro lado, solo se incluyeron pacientes con cristales de OCD, OCM y AU que cumplían con los criterios descritos, pero estrategias de prevención de CN más completas deberían incluir pacientes con otros cristales urinarios como brushita, estruvita o cistina. Finalmente, dado que no se ha registrado el grado de cumplimiento de las medidas higiénico-dietéticas aplicadas por cada paciente, no es posible descartar la posibilidad de que alguno de ellos no siguiera las pautas descritas, lo que podría subestimar los resultados obtenidos. En base a este hecho, los resultados presentados en este trabajo pueden considerarse preliminares y estudios futuros deberán tener en cuenta estas limitaciones para obtener información más detallada sobre el efecto de aplicar medidas higiénico-dietéticas para la reducción del número de CN, siendo importante la evaluación del grado de fidelidad con el que cada paciente incorpora las recomendaciones proporcionadas a través de un seguimiento individualizado periódico.

## Conclusiones

La introducción de mensajes que alertan sobre el riesgo litogénico junto con la inclusión de medidas higiénico-dietéticas en los informes de laboratorio son herramientas para tratar de reducir la incidencia de CN.
